# School-Based and PRECEDE-PROCEED-Model Intervention to Promote Physical Activity in the High School Students: Case Study of Iran

**DOI:** 10.5539/gjhs.v8n9p271

**Published:** 2016-01-31

**Authors:** Baratali Rezapour, Firoozeh Mostafavi, Hamid Reza Khalkhali

**Affiliations:** 1 Department of Health Education & Health Promotion, Isfahan University of Medical Sciences, Isfahan; 2 Department of Public Health, School of Health, Urmia University of Medical Sciences, Urmia, Iran; 3 Inpatient’s Safety Research Center, Urmia University of Medical Sciences, Urmia, Iran

**Keywords:** PRECEDE- PROCEED- Model, School-based intervention, physical activity

## Abstract

**Objectives:**

Students attend sedentary life style and less like vigorous physical activity. This study investigated the effects of School-based intervention on increasing physical activity for decreasing obesity among high-school obese and overweight boys, based on the components of PRECEDE PROCEED Model, to participate in median - vigorous physical activity among the first Period of high school boys in the city of Urmia, Iran

**Methods:**

This study was an experimental intervention that conducted at 4 high schools that were divided into 2 groups of intervention (40) and the control (40) male students, schools in junior high schools in Urmia.

**Results:**

Three and six months after the intervention, significant differences were found between the experimental and control groups of schools, in the amount of students’ participation in vigorous physical activity (p<0.01).

**Conclusions:**

According to the results, the school-based intervention and components of PRECEDE PROCEED Model had a positive impact on the improvement of physical activity and decrease in physical inactivity among the students.

## 1. Introduction

The last demonstrative increase in the rates of obesity in students indicates that there is an urgent necessity for the schools to consistently and efficiently advance physical activity PA manners that will protect the students from overweight and obesity. PA is a determinative key of weight situation. Increasing “screen time” and decreasing confidence on PA transportation suggest that schools should assume a management role in ensuring that students engage in adequate amounts of PA each day (Russell et al., 2006). The significance of PA in decreasing prevalence of overweight and obesity and also illness and death from ailment has been well established (Jafari-Adli, Jouyandeh, Qorbani, Soroush, Larijani, & Hasani-Ranjbar, 2014). In that time we must consider a significantly expanded role for schools in providing PA to students. School staffs must help students to participate in PA to create PA lifestyle (Daniels et al., 2005). The main idea of this paper is PA. It states the important roles of schools about PA. Interventions like SBI and theoretical health education based on PRECEDE -PROCEED-Model (PPM) programs can increase time of PA per week and decrease the prevalence of overweight and obesity (Yarahmadi et al., 2013). Diabetes, stroke, heart disease, and cancer are causes for two-thirds of mortality. Sedentary life style and lack of vigorous physical activity (VPA), cause overweight and obesity that often establish in childhood; 33% of students attended daily physical education (PE) classes and 72% of students did not participate; So the school district should support PA and provide school environments to promote and protect students’ health, well-being, and ability to learn (Activity., 2005). The national prevalence of obesity in Iran was seen significantly different in the sub-national prevalence (Jafari-Adli et al., 2014). Sedentary life style (SLS) causes 310,000 to 580,000 deaths per year. Studies showed that 14% of all U.S. deaths in 1990 (Jafari-Adli et al., 2014) and 23% of chronic disease-related deaths in the U.S. in 1986 could be attributed to poor activity pattern and SLS (Roya, Gelayol, Mostafa, Asal Ataie-Jafari, Maryam Bahreynian, Mahnaz Taslimi, Mohammad Esmaeil Motlagh, & Heshmat, 2013). Regular PA is essential for a healthy life (Yarahmadi et al., 2013). SLS has caused obesity and overweight in people increase rapidly over the past few years in Iran. Recess breaks have been reduced in some high schools (Waite-Stupinsky, 2001). More than one third (38.2%) of students spent > 3 hours per day watching television (Grunbaum et al., 2005). High school students’ participation in daily Physical education (PE) classes decreased since 1991 to 2003 (Prevention., 2004). Walking or biking to school has decreased (Prevention., 2005; Services., 2000). School environments and curricular and extracurricular school programs and parents monitoring promote positive health behaviors such as increase in PA, reduce SLS and watching television, because students spend large amounts of time in the schools (McKenzie et al., 1996). The global school-based health survey (GSHS) in students has been developed by World Health Organization (WHO) and Centers for Disease Control and Prevention (CDC) in cooperation with United Nations’ UNICEF, UNESCO, UNAIDS. The “childhood and adolescence surveillance and prevention of adult non-communicable disease” (CASPIAN) is a national school-based surveillance of the risk behaviors of chronic diseases using GSHS among students in Iran (Roya et al., 2013). Over the past decade, several organizations have recommended that students participate in ≥60 minutes of PA each day in school (Strong et al., 2005). There were few programs to manage or prevent overweight and obesity for students in Iran. The last increase in students’ overweight and obesity rates needs that the schools address this problem by adopting policies that require daily PE, school recess, and PA opportunities before, during, and after school (Policies., 2005). This research was used PPM to intervention in students and schools to change present situation. The key components and constructs of PPM are: Enabling Factors (EF), Predisposing Factors (PF) (include knowledge and attitudes and self-efficacy) and Reinforcing Factors (RF). In this study, educational diagnosis explored those three factors. PF are the individuals’ or populations’ knowledge, attitudes, that facilitate or inhibit health behaviors (Li, Cao, Lin, Li, Wang, & Jia, 2009). And the Questionnaire of Baecke et al Questionnaire is the most common PA assessment tool used in studies about obesity or overweight, evaluating students’ PA and separating it into three distinct dimensions: work activity (WA), sports activity (SA) and leisure activity (LA) (Baecke, Burema, & Frijters, 1982). It has not yet been demonstrated whether SBI can reduce BMI or the prevalence of overweight and obesity, so additional research is needed to clarify the relationship between in-school and out-of-school activity.

School staffs should teach parents and other family members about benefits of regular PA, to help all family members participate in PA, and also help students to create a positive attitude toward PA, and participate in child care, food preparation, and gardening, house cleaning, and shopping as a basic component of coping with overweight and obesity. School staffs and teachers should encourage positive attitudes toward PE, introduce the principles and methods of PA and provide opportunities for students to learn exercise skills to prepare for PA. The school curriculum should include all of the students. School staffs and teachers should also teach the benefits of PA and the development and maintenance of PA conditioning throughout life (Fletcher et al., 1992). In order to present both information about PA and exercise and general information, the intervention educational approaches were developed (e.g., ways to decrease the risk of BMI>25 and its Complications). The information is intended to change students’ knowledge and attitude about the benefits of PA, explain methods for overcoming barriers and negative attitudes about PA, and ultimately increase PA. This paper describes the results of a study that examines whether the application of educational SBI can be used effectively to change the perception of the junior high school students on the components of PPM such as (EF, RF, PF) and about PA and work index and sport index and leisure index and changing SLS, in order to increase PA among the junior high school students in Urmia.

## 2. Methods

### 2.1 Design and Setting

This was an experimental interventional SBI study that was carried out in two districts of Urmia, Iran, since December 2013 to January 2014. After obtaining permission from the Isfahan University of Medical Sciences (IUMS) and from the Provincial Education Department, the researcher entered the schools. Schools were as the unit of randomization and analysis. Percentage of students who were obese or overweight was 7. Class was held for 60 minutes for each session. Schools from each pair were randomly allocated to control or experimental groups; research results were assessed with data from school-based samples of students. The intervention was developed to modify both the educational program and the school environment, and all seventh-grade obese or overweight students who had enrolled in intervention school were subjected to the intervention. Data collection was performed in 3 periods. Baseline measures and 3 and 6 month (Follow-up) measures after exposure to the intervention occurred during the seventh grade. In this study comparisons took place between selected PA variables and 2 experimental and control schools, and additional analyses were performed to examine the effect of PA during PE classes on height and weight and BMI of students.

### 2.2 The Population of Study

All seventh-grade students (N=80) who were obese or overweight and attending in the high-school classes in intervention and control schools were invited to complete the questionnaires. Incentives for the intervention group (n= 40 obese or overweight students) who participated in briefing classes were described (sports shirt). The control group (n= 40 obese or overweight students) from the selected high schools completed baseline questionnaire. Each student was provided written informed consent before data collection. The students were informed that all the obtained data were used without personal identifiers and were therefore confidential.

### 2.3 Intervention

The intervention was projected to change both the educational practices and the school environment (SE) to enhance support for PA among students and also it was projected on the basis of PPM. A guide pamphlet based on components of PPM and school-based intervention (SBI) provided for participants. The components of SBI include physical education (PE), School health services technician, SE, buffet school, teachers association, and parent–teacher association (PTA). SBI activities were coordinated by school PE teacher. School-based PE was carried out by practical implementation and oral explanation. After completion of the questionnaire by both groups, the educational program was performed in the experimental group and educational intervention was directly conducted through lecture, collaborative methods and group discussion and role playing in six sessions (60 minutes per session). The PE intervention was implemented by changing the content of PE and specific teaching of the physical and behavioral skills needed for obese and overweight students to involve them in moderate-to vigorous physical activity (MVPA) during PE class time. PE must include specific student’s choice-based sports to reinforce participation in PA. Activities that students typically enjoy (e.g., football, volleyball, ping pong, cycling) were implemented in addition to competitive sports and other traditional PE activities. The environmental intervention was designed to create a SE that supported PA among students. Environmental change activities included preparation volleyball, football, ping pong and cycling tools, and the other important change to increase communication between obese and overweight students about PA and sports by development sports team with obese and overweight students, and promotion of PA by the school health staff, and PTA.

### 2.4 Measurement of Physical Activity

Data were collected by trained interviewers. They explained the intensity level of common activities. Participants were asked to complete the questionnaire. The data were collected by the same questionnaire three times (before, three and six months after intervention, follow-up). The SBI and data collection instrument was a 3-part questionnaire; the first part of questionnaire includes Demographic characteristics of the participants like age, father literacy (FL), mother literacy (ML), Occupation of father (OF), Occupation of mother (OM), Family size (FZ), Obesity in the family (OIF), Anthropometric measurements by quantitative techniques for determining students individual’s BMI by measuring height and weight, and the second part was a PPM-Based Questionnaire that included the components constructs of PPM such as: Enabling Factors (EF) (α=0.791), Predisposing Factors (PF) (include knowledge (α=0.754) and attitudes (α=0.729) and self-efficacy (α=0.836)) and Reinforcing Factors (RF) (α=0.776). The third part of the questionnaire was Baecke et al. Questionnaire that included Work Activity (α=0.726), Sport Index (α=0.675), Leisure Index (α=0.881). The Baecke included 16 items, with proven validity in previous studies about information on tested (Baecke, Burema, & Frijters, 1982; Mirzaee Vishgaee, Rahmani Niya, & AR, 2014; Ono et al., 2007; Peter & Bandmann, 2008; Saris, Snel, Baecke, van Waesberghe, & Binkhorst, 1977; To fighi, S. Babaei, Eloon Kashkuli, & R. Babaei, 2014). The Baecke questionnaire was used to assess PA of participants in each schools and it was determined on the basis of the typical intensity range for that activity adjusted for the subject’s intensity rating (light, moderate, and very hard). Weight of participants was measured with light clothing to the nearest 100 gram and height was measured using a stadiometer. Then BMI was calculated from weight (in kg) divided by a square of the height (in meters). The questionnaires evaluated the effectiveness of the SBI and PPM-based educational interventional programs about PA and prevention and reduce obesity and overweight in students.

### 2.5 The Outcome of Variables

The aim of intervention was promoting PA participation in other settings and increasing the intensity and the duration of PA during PE classes and obtaining students PA guidelines. The other aim of the intervention was at increasing of students on components of PPM such as: predisposing, reinforcing and enabling factors scores about PA. The researcher hypothesized that significant increase occur in the intervention participants on regular vigorous physical activity (RVPA). Because of concerns about the rising prevalence of obesity, the prevalence of overweight and at-risk for overweight also was treated as secondary outcome of variables.

### 2.6 Statistical Analysis

Means and standard errors were computed for the PA variables at seventh grade baseline and at follow-up (three and six month after intervention). To determine the effects of the intervention, PA variables and weight categories was analyzed for both the intervention and the control groups with repeated measured analysis of variance (RM.ANOVA). Because the students were from 4 different high schools, and because students within a school share a unique social and physical environment, the statistical analysis was designed to control for the influence of school. In this study independent t-test was used to test the significance of the difference between students in intervention versus control schools for the percentage of RVPA. This analysis was repeated after 3 and 6 month for percentage of RVPA among students at follow-up was imputed by applying an RM.ANOVA to the available data.

## 3. Results

There were no significant differences in the demographic variables (such as age or parents’ Literacy, and Job, Family size and Obesity of parents, Brother, Sister, Uncle, Aunt, Grand-mother and Grandfather) between students in the control and intervention schools (Table 1). Also Students were the same age (12-14 years) and were similar in BMI at baseline in the control and intervention schools. All (80) students who were measured at baseline also were measured at follow-up. At 3 and 6 month after intervention (follow-up), the R.M.ANOVA showed that the regular VPA was greater in the intervention schools than in the control schools (P<0.001). About 49.3% of students in the intervention schools and 41.6% of students in the control schools had participated in VPA. At baseline the intervention and the control schools were not significantly different (Table 2). Approximately 75% of students in the intervention and 32.5% in the control schools were participated more than 4 hour per week in PE classes as seventh-grade. Replication of the outcome analysis with the subset of students who participated in PE classes moderately increased the efficacy of the intervention, with 77.5% of students in the experimental schools and 57.5% of students in the control schools meeting the VPA standard (P<0.001). The percentage of students who at baseline were categorized as overweight or at-risk for overweight (BMI≥25); was 20%. After 6 months approximately 22.5% of students were classified as overweight; and after 6 month approximately 7.5% of students have decrease in BMI (25-30), but there were no significant differences between the intervention and the control schools.

**Table 1:** Frequency Distribution of the Demographic Characteristics of Students at baseline in Urmia 2013- 2014

variable	group	Experimental group		Control group		p-value
	
		No	%	No	%	
Students’ Age	12	11	27.5	12	30	0.895
	13	14	35	15	37.5	
	14	15	37.5	13	32.5	
father’s Literacy	3	23	57.5	18	45	0.50
	4	17	42.5	22	55	
Mother’s Literacy	3	21	52.5	16	40	0.262
	4	19	47.5	24	60	
father’s job	1	16	40	19	47.5	0.499
	2	24	60	21	52.5	
mother’s job	1	9	22.5	6	15	0.576
	1	31	77.5	34	85	
Family size	≥4	34	85	32	80	0.556
	5≤	6	15	8	20	
Father’s Obesity	yes	21	52.5	23	57.5	0.653
	No	19	47.5	17	42.5	
Mother’ s obesity	yes	26	65	31	77.5	0.217
	No	14	35	9	22.5	
Brother’s obesity	yes	33	82.5	35	87.5	0.531
	No	7	17.5	5	12.5	
Sister’s obesity	yes	39	97.5	36	90	0.166
	No	1	2.5	4	10	
Uncle’ s obesity	yes	31	77.5	27	67.5	0.317
	No	9	22.5	13	32.5	
Aunt’s obesity	yes	34	85	36	90	0.499
	No	6	15	4	10	
Grandmother’s obesity	yes	25	62.5	30	75	0.228
	No	15	37.5	10	25	
Grandfather’s obesity	yes	29	72.5	33	82.5	0.284
	No	11	27.5	7	17.5	

**Table 2:** Comparison of the Mean Scorers Components of PPM about PA in the 2 groups in the before, 3 months after and 6 months after Intervention

(PPM) Components Variables	Analysis		before Intervention		3 months after Inter		6 months after Intervention	

			Mean	SD	Mean	SD	Mean	SD
PREDISPOSING Factor	knowledge	Experimental	5.47	(1.46)	7.35	(1.23)	7.85	(1.57)

		Control	5.17	(1.93)	6.50	(2.07)	7.45	(2.06)

		P-value	P=0.437					

	Attitude	Experimental	20.02	(3.44)	20.80	(1.91)	22.67	(1.61)

		Control	19.15	(2.71)	17.6	(2.18)	18.20	(1.95)

		P-value	P=0.21					

	Self-Efficacy	Experimental	15.125	(3.13)	16.5	(1.79)	17.05	(1.95)

		Control	13.52	(2.93)	11.80	(2.10)	13.02	(2.15)

		P-value	P=0.21					

ENABLING FACTOR		Experimental	11.95	(2.54)	23.55	(1.97)	24.80	(0.40)

		Control	11.25	(2.01)	21.98	(1.73)	22.25	(1.45)

		P-value	P=0.176					

REINFORCING FACTOR		Experimental	3.725	(1.04)	5.87	(1.34)	6.97	(1.33)

		Control	3.45	(1.29)	3.77	(1.21)	4.15	(1.98)

		P-value	P=0.29					

Table 1 demonstrates the frequency distribution of the demographic characteristics (Students’ Age, father’s Literacy, Mother’s Literacy, Father’s job, Mother’s job, Family’s size, Father’s Obesity, Mother’s obesity, Brother’s obesity, Sister’s obesity, Uncle’s obesity, Aunt’s obesity, Grandmother’s obesity, Grandfather’s obesity) of the two groups of students. There was no significant statistical difference between demographic variables such as: Students’ Age (P=0.895) father’s literacy (p=0.50) mother’s literacy (p= 0.262) in the intervention and control groups.

Table 2 compares the two groups’ (intervention and Control) mean scores of Components of PPM constructs (Predisposing Factor (that constructs from knowledge, attitude and Self-Efficacy) Enabling Factor and Reinforcing Factor in three phases of before intervention, three and six months after the intervention. There were no significant differences between the two groups in PPM constructs (PF, EF and RF) at baseline, but three and six months After the SBI intervention, a significant increase was found in the mean score of each Components of PPM of students in the intervention group (p<0.001), compared with the baseline for PPM constructs such as: PF(knowledge P=0.437, attitude P=0.21, Self-Efficacy P=0.21), EF P=0.176, RF P=0.29, Furthermore, in the baseline, there was no significant differences between RF, PF, EF of the students in the interventional and control groups; however, in the post-intervention time (three and six months after the intervention) R.M.ANOVA showed a significant difference between the intervention and control groups in terms of the mean score of each Components of PPM constructs (p<0.001). The mean and standard deviation of the knowledge in the two groups (Table 2) R.M.ANOVA showed a significant difference between the knowledge in the intervention and control groups (F(1, 78) = 7.96, P<0. 01, Partial Eta Squares = 0.09). The mean and standard deviation of the knowledge in the two groups R.M.ANOVA showed (Table 2) that the intervention was significant (F(2, 156) = 12.38, P<0.001, Partial Eta Squares = 0.14), and the interaction between the time trend is significant intervention F(2, 156) =40.37, P<0.001, Partial Eta Squares = 0.34). The mean and standard deviation of the Attitude in the two groups (Table 2) R.M.ANOVA showed that the intervention was significant (F(1, 78) = 52.07, P<0. 001, Partial Eta Squares = 0.40). The main effect of time, F(2, 156) = 8.37, P<0.001, Partial Eta Squares = 0.10) and the interaction between the time trend is significant intervention F(2, 156) =17.40, P<0.001, Partial Eta Squares =0.18).

The mean and standard deviation of the SE in the two groups R.M.ANOVA showed (Table 2) that the intervention was significant (F (2, 156) = 5.58, P=0.005, Partial Eta Squares = 0.07).

The main effect of time, F (2,156) = 16.8, P<0.001, Partial Eta Squares = 0.18) and the interaction between the time trend is significant intervention F (1.78) =65.1, P<0. 001, Partial Eta Squares =0.45.

The mean and standard deviation of the EF in the two groups (Table 2) R.M.ANOVA showed that the intervention was significant (F (1, 78) = 38.7, P<0.001, Partial Eta Squares = 0.33.

The main effect of time, F (2,156) = 1.56, P<0.001, Partial Eta Squares = 0.95) and the interaction between the time trend is significant intervention F (2,156) =7.5, P=0.001, Partial Eta Squares =0.09.

The mean and standard deviation of the RF in the two groups (Table 2) R.M.ANOVA showed that the intervention was significant (F (1, 78) = 67.05, P<0.001, Partial Eta Squares = 0.46.

The main effect of time, F (2,156) = 87.8, P<0.001, Partial Eta Squares = 0.53) and the interaction between the time trend is significant intervention F (2,156) =38.04, P<0.001, Partial Eta Squares =0.33.

Table 3 compares the two groups’ (intervention and Control) mean scores of Components of PA constructs Work Activity (WA), Sport Activity (SA) and Leisure Activity (LA) in three phases of before intervention, three and six months after the intervention. BMI in the 2 groups in the baseline and 6 months after Intervention (Follow up) was investigated. 6 month after the SBI, a significant increase was found in the mean score of each components of PA of students in the intervention group compared with the baseline, while there were no significant changes in the mean score of the mentioned variables, before, three and six months after the intervention, in the control group; however, in the follow up, R.M.ANOVA showed a significant difference between the intervention and control groups in terms of the mean score of each components of PA constructs WA (3 and 6 month period p<0.001), SA(Sport active 3 and 6 month period p<0.061). The mean and standard deviation of the WA in the two groups (Table 3) R.M.ANOVA showed that the intervention was significant (F(1, 78) = 28.9, P<0.001, Partial Eta Squares = 0.27). The main effect of time, F(2,156) = 23.13, P<0.001, Partial Eta Squares = 0.229) and the interaction between the time trend is significant intervention F(2,156) = 2.33, P=0.1, Partial Eta Squares = 0.03). Table 3 also compares the two groups’ (intervention and Control) mean scores of BMI in two phases of before intervention and six months after the intervention. Although BMI reduced in three and six months after intervention, there were no significant differences between the two groups in BMI before and six months after the intervention. (P=0.526).

**Table 3:** Comparison of the Work Activity, Sport Activity and Leisure Activity and BMI for Prevention and treatment of obesity and overweight in the 2 Groups in the Pre and 3 and 6 months after Intervention

Physical activity Components Variables	Analysis	baseline		3 months after Inter		6 months after Inter	

		Mean	(SD)	Mean	(SD)	Mean	(SD)
Work Activity WA	Experimental	3.47	0.47	3.43	0.49	3.21	0.54

	Control	3.19	0.41	2.99	0.34	2.69	0.40

	P-value	P=0.06					

Sport Activity SA	Experimental	3.06	1.38	3.13	0.97	4.75	2.19

	Control	3.15	1.59	3.28	1.52	3.76	2.44

	P-value	P=0.794					

Leisure Activity LA	Experimental	12.42	3.44	10.85	3.94	11.37	3.36

	Control	11.07	3.44	10.75	3.45	9.95	2.65

	P-value	P=0.70					

Body Mass Index	Experimental	27.70	3.42		27.19		3.04

	Control	27.94	3.08		27.65		3.37

BMI	P-value	P=0.749					

## 4. Discussion

This study is the first to show that a SBI can increase regular participation in VPA among high-school students. After one educational year of exposure to a theory based PA intervention, the percentage of students who reported regular VPA was approximately 7.9% greater in the intervention schools than in the control schools (49.3% vs. 41.4%). This study shows that school programs can promote PA among students. Many public health authorities have identified promotion of PA as a critical strategy for conflict with SLS obesity and overweight (Russell et al., 2005). This study targeted all obese or overweight seventh-grade students who attended the intervention schools. The results of study show that the overall distribution for VPA changed (32.5% to 47.5) to a higher level among students who were in the experimental schools. This shift, although not significant, is important because it applies to all of the students who were exposed to the intervention (Russell et al., 2005). In addition, some teachers in the PE classes sometimes give their students football or volleyball Ball, and students didn’t learn the principles and skills of sports. Some students have no interest in the football or volleyball and they will stand aside and be just a spectator. On the other hand some of the students’ interests are basketball or ping pong or other sports in school PE classes, but teachers do not teach those needed skills, so they don’t participate in school PE programs. Using school-based theoretical health education and health promotion is effective method to increase PA of students in schools. The results of the present study revealed that prior to the intervention, many components of PPM (EF, RF, and PF, (that is constructed in the basis of knowledge, attitude and SE) were below average in baseline among interventional and control groups. After the intervention, a significant improvement was found in the behavior of the participants of the experimental group, while only a slight change was observed in the behavior of the participants of the control group. These findings support our hypothesis that SBI and health education programs based on PPM can be effective in improving the PA of students. Several studies have identified some basic educational needs of the individuals, which increase their PPM components, leading to promotion of PA. The findings of the present study indicated that components of PPM (EF, RF, and PF) scores of the participants on PA significantly increased after the intervention in the experimental group. Education based on framework of the PPM is effective to increase the score of PF (knowledge, attitude, and self efficacy), EF and RF. These results are similar and consistent with the findings of the study conducted by Zigheymat F. et al. (2009), Jafari-Adli et al. (2014). Given the prevalence of overweight and obesity and the associated morbidity and mortality, it is important to increase the knowledge and attitude and self efficacy of PA and encourage the adoption of PA that help prevent this condition as opposed to waiting until the onset of the overweight and obesity. The results of this study revealed that school-based health education programs based on PPM interventions focusing on increasing PA are ideally suited to reach the goals of increased knowledge, and adoption of school-based health education programs about PA. The differences between the mean scores of the experimental group on the PA were significant after the intervention (p< 0.01).

The increase in the performance of the participants in this study is also consistent with the findings of Jafari-Adli et al. (2014), and Tell et al. (Tell, & Vellar, 1988) and Killen et al. (Killen, Telch, Robinson, Maccoby, Taylor, & Farquhar, 1988) and Robinson et al. (Russell et al., 2006; Robinson, 1999) and Epstein et al. (Epstein, Saelens, Myers, & Vito, 1997; Smith, Vara, & Rodefer, 1991; Russell et al., 2006) and Ford et al. (Ford, McDonald, Owens, & Robinson, 2002; Russell et al., 2006). Although BMI was reduced in three and six months after intervention but there were no significant differences between the two groups in BMI before and six months after the intervention. (P=0.526)

## 5. Conclusion

Considering the poor EF, RF, PF (knowledge, attitude, Self-Efficacy), and performance of the students about the PA and the positive effect of SBI and theoretical-based such as PPM education on this matter, it seems that this type of education can supply the necessary grounds for enhancing the PPM components and performance of the students and the society. Besides, considering the important role of students as the future adults and the low cost of preventive measures such as PA education as compared with the treatment measures of obesity and overweight, it seems necessary to develop such educational programs among other related groups and populations especially Iranian students.

Enormous potential appears to exist for schools to expand their role in providing students with additional PA by building institutional relationships with school-based providers of PA. Schools can make their facilities available to community-based organizations during after-school, weekend, and summer periods. Also, schools can collaborate with community organizations in transit authorities, and promote PA programs to students and their parents.

**Moral Matters**

Ethical matters have been wholly intended by the authors.
